# A Syringe in an Emergency Saves Time: An Audit on the Utility of Syringe Provision in Eye Emergency

**DOI:** 10.7759/cureus.37618

**Published:** 2023-04-15

**Authors:** Hamna Zafar, Redaina Akhtar, Aruba Zafar, Umema Zafar

**Affiliations:** 1 Ophthalmology, Khyber Teaching Hospital, Peshawar, PAK; 2 Ophthalmology, Shifa Hospital, Islamabad, PAK; 3 Physiology, Khyber Medical University, Peshawar, PAK; 4 Physiology, Rehman Medical College, Peshawar, PAK

**Keywords:** corneal trauma, foreign body, syringe, eye emergency, audit

## Abstract

Background

Patient satisfaction is the top priority of health care facilities in addition to the quality of health care delivery services. The convenience of health care receivers, be it temporal or monetary, fall in this domain. Hospitals should be equipped with dealing with all kinds of emergencies no matter how trivial or catastrophic.

Aim

To improve the provision of emergency care equipment (1 c.c. syringes) in the examination room of our ophthalmology department by 50% in two months’ time.

Materials and methods

This quality improvement project (QIP) was conducted in the ophthalmology department of a teaching hospital in Khyber Pakhtunkhwa. This QIP was conducted over a period of two months in the form of three cycles. All cooperative patients with embedded and superficial corneal foreign bodies who presented to the eye emergency were included in the project. The provision of 1 c.c. syringes in the emergency eye care trolley of the eye examination room was ensured at all times after the first cycle survey. A record was maintained of the percentage of patients receiving syringes from the department as well as the percentage of patients purchasing them from the pharmacy. The progress was measured every 20 days, following the approval of this QI project.

Results

A total of 49 patients were included in this QIP. This QIP shows that the provision of syringes was improved to 92.8% and 88.2% in cycles 2 and 3 from the previous statistics of 16.6% in the first cycle.

Conclusions

It is concluded that this QIP achieved its target. The provision of emergency equipment, such as a 1 cc syringe costing less than 1/20th of a dollar, is a simple act that saves resources and improves patient satisfaction.

## Introduction

Corneal abrasions and foreign bodies on the ocular surface are two of the most common ophthalmic complaints of patients presenting to the emergency departments [1]. Some ocular complaints, although not emergent, can cause extreme discomfort to the patient, whereas other problems can be vision threatening and require immediate ophthalmological consultation [2]. The cornea is richly innervated, hence corneal foreign bodies can present with symptoms like pain. Symptoms typically occur acutely and include foreign body sensation, decreased vision, photophobia, increased tear production, blepharospasm, and conjunctival injection [3]. Foreign body sensation, which can be felt as a gritty sensation in the eye is one of the most common presenting complaints. For patients who complain of foreign body sensation, a complete history and eversion of both eyelids are necessary to rule out a foreign body lodged in the fornices and subconjunctival space [4]. Commonly occurring foreign body substances include metals, glasses, eyelashes, wood, stone, larvae, wings of insects, beans, plants, and other organic materials [4,5]. Conjunctival foreign bodies are usually found in the sub-tarsal sulcus. The possibility of a superior forniceal subconjunctival foreign body should be considered in patients presenting with itching, foreign body sensations, and multiple corneal abrasions [4].

The cornea and the lens are responsible for focusing light falling on it onto the retina. Corneal foreign bodies can subsequently lead to corneal edema, thereby disrupting vision and causing scarring and irregularities [3]. Hence, the timely removal of a corneal foreign body is essential to restore vision and prevent infection and scarring. As emergency departments are usually staffed with junior doctors, they are the ones required to attend to patients presenting with ophthalmic foreign bodies [6]. In the UK, most patients presenting with ocular complaints to the emergency departments are seen by foundation year two doctors who were previously known as senior house officers [7]. Materials that are tolerated by the patient may be left in place with subsequent ophthalmic follow-ups. However, poorly tolerated objects need prompt removal [8]. Some of the equipment required for the removal of ophthalmic foreign bodies used in emergency departments, urgent care centers, primary care, and eye care providers include topical sodium fluorescein, topical anesthetic drops (proparacaine 0.5% or tetracaine 0.5%), slit-lamp with a cobalt blue filter or a Burton lamp with a similar filter, sterile saline, sterile cotton-tipped applicators, jewelers forceps, a 25 gauge needle attached to a tuberculin syringe (a bent needle tip is optional), foreign body spud (consider a magnetic spud for metallic foreign bodies), rotating burr tool (Alger brush), and an eyelid speculum [3]. In patients complaining of ocular foreign bodies, eye irrigation can be done after excluding open globe injuries. External irrigation can wash out retained foreign bodies in some cases. Among these patients, this procedure can greatly diminish pain and on further examination, foreign bodies are uncommonly seen [1]. A slit lamp is used to magnify the anterior segment of the eye as well as illuminate it. It is also required to position and immobilize the patient during the procedure [9]. A syringe needle is usually used to remove embedded corneal foreign bodies presenting to the emergency department. Less commonly, an eye spud may be used. A microneedle holder can be used as a substitute for an eye spud or syringe needle to remove foreign bodies from the cornea under slit lamp examination. A needle syringe can increase the risk of corneal penetrating injury, especially in patients who are uncooperative. Microneedle holder in this regard is a relatively safer instrument [10]. Full thickness corneal or intraocular foreign bodies may require surgical intervention [11].

Emergency care provision is a fundamental right for all patients presenting in an emergency to the hospital. An emergency care trolley should be sufficiently stocked with 1 cc syringes so that patients can be spared the trouble of going to the pharmacy and purchasing syringes. The purpose of this quality improvement project (QIP) was to improve the provision of out-of-hours emergency care equipment (1 cc syringes) in the eye examination room in the Ophthalmology B department of Khyber Teaching Hospital Peshawar by 50% in two months’ time.

## Materials and methods

This quality improvement project (QIP) was conducted in the Ophthalmology B department at Khyber Teaching Hospital from November 1, 2021, to December 31, 2021. The key staff groups involved in this project were doctors, nurses, and pharmacists. Ethical approval for this QIP project was obtained from the institutional research and ethical board with reference No.1031/DME/KMC dated November 1, 2021.

**Figure 1:**
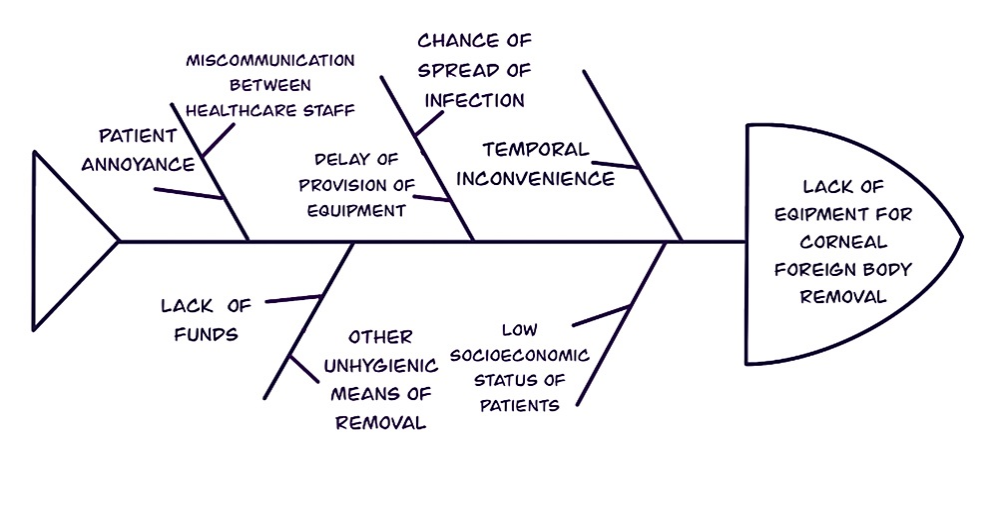
Ishikawa diagram showing the need for PDSA PDSA: plan-do-study-act

Figure 1 presents an Ishikawa diagram to show the need for PDSA.

**Figure 2:**
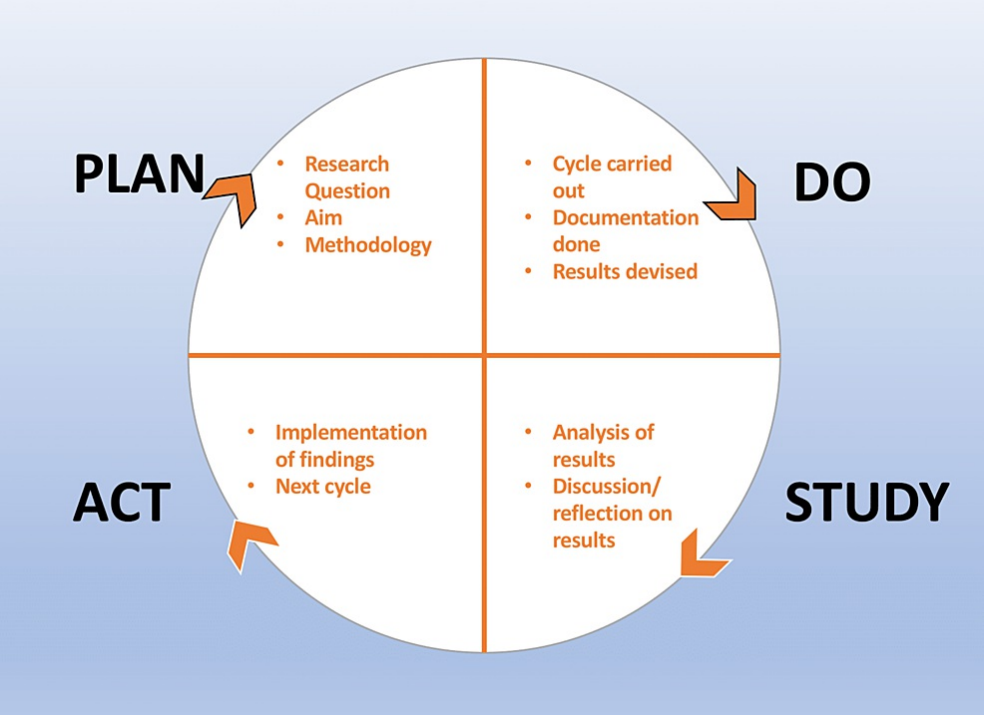
Stages of PDSA This figure is based on the general PDSA process customized by the authors according to the current project. PDSA: plan-do-say-act

This QIP was composed of three cycles. The duration of the first, second, and third cycles was 20 days each. This QIP was conducted as a plan-do-study-act (PDSA). PDSA is a four-stage problem-solving model used for improving a process or carrying out change (Figure 2).

During the first cycle, a survey was done before the provision of equipment to document the percentage of individuals who had to buy syringes for the removal of foreign bodies. There was no intervention done in the first cycle; it consisted of a preliminary survey for gauging the need and the actual situation regarding the syringe provision. The second cycle onward involved contacting the hospital pharmacy which supplies pharmacological equipment to all departments and requesting them to make an arrangement for 1cc syringes for the removal of foreign bodies from the eye. Subsequently, the nursing staff responsible for making the essential stock list for the Ophthalmology department were instructed to add 1 cc syringes to the list, which was then forwarded to the pharmacy on a weekly basis. In the eye examination room, the emergency eye care trolley was adequately stocked with 1 cc syringes for immediate foreign body removal at the slit lamp.

The further process involved receiving patients at the emergency where they were directed to the concerned department. Upon arrival, patients were examined by the house officer on duty. If the patient required the removal of a foreign body via a 1 cc syringe, it would be available in the emergency trolley of the ophthalmology department rather than sending the patient to a pharmacy outside the hospital to buy equipment. A record was maintained of the percentage of patients receiving syringes from the department as well as the percentage of patients purchasing them from the pharmacy. The progress was measured every 20 days, following approval for this QI project. This was continued for three cycles, after which a final report was generated. For sustenance of results, a house officer was delegated from the next rotation of doctors for maintaining 1cc syringes as a permanent addition to the list of departmental product supplies.

Inclusion criteria involved embedded and superficial corneal foreign bodies. Those superficial foreign bodies that were adhesive but not embedded and were unable to be removed via noninvasive procedures like saline irrigation were also included. All ages that were cooperative and able to consent to the procedure were included in the process.

 Intraocular foreign bodies and superficial foreign bodies that could be removed with saline irrigation, forceps, or sterile cotton-tipped applicators were excluded. Uncooperative patients and those unwilling to consent were excluded from the project.

## Results

The first cycle of this study included 18 participants. Out of these, 13 (72.2%) were males and five (27.7%) were females. Participants from ages 0 to 10 were one (5.50%), from 11 to 20 were seven (38.80%), from 21 to 30 were four (22.22%), from 31 to 40 were four (22.22%), and from 41 to 50 were two (11.11%). The second cycle included 14 participants. Out of these, 13 (92.8%) were males and one (7.14%) was female. Participants from ages 11 to 20 were eight (57.14%), from 21 to 30 were 4(28.57%), and from 31 to 40 were two (14.28%). The third cycle included 17 participants. All the participants were males. Participants from ages 11 to 20 were two (11.76%), from 21 to 30 were 10 (58.82%), from 31 to 40 were one (5.88%), from 41 to 50 were three (17.64%), and from 51 to 60 were one (5.88%).

The given graphs illustrate the percentages of people that bought syringes for foreign body removal, as well as those who did not have to buy syringes due to availability in the eye examination room. Overall, during the first survey, most people had to buy syringes from a pharmacy, whereas, during the second and third cycles, the examination room was equipped with syringes so fewer people had to purchase them.

**Figure 3:**
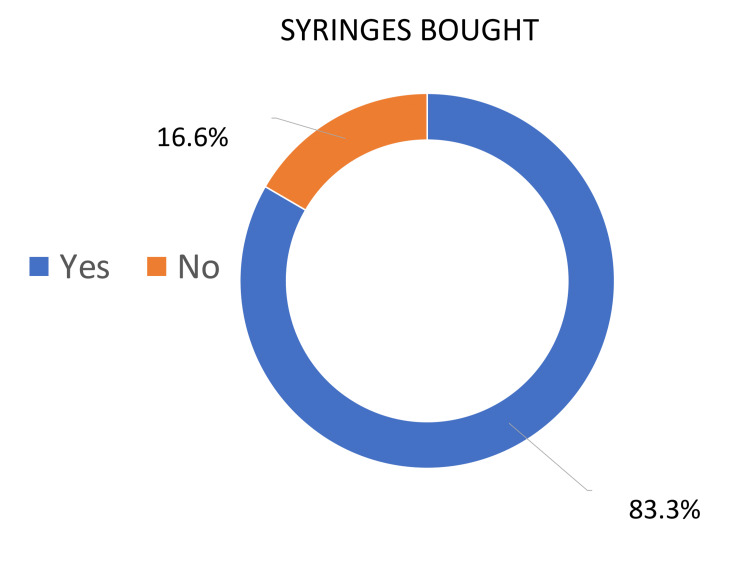
Cycle 1

**Figure 4:**
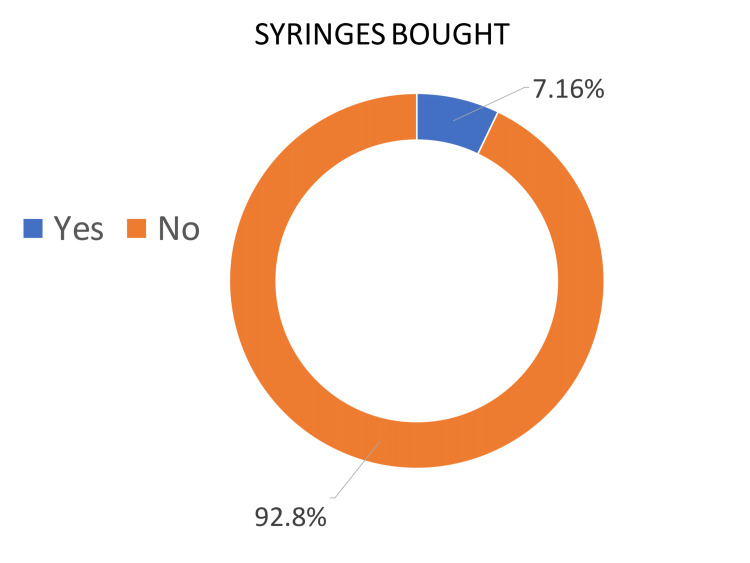
Cycle 2

**Figure 5:**
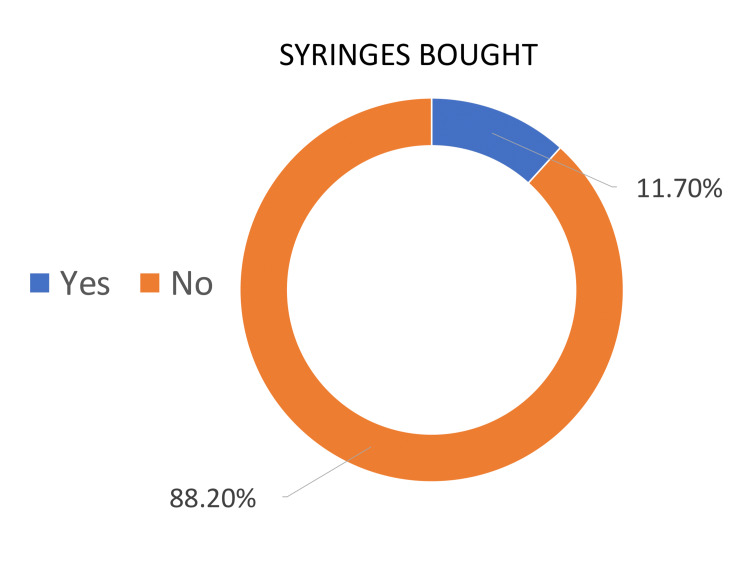
Cycle 3

Cycle one demonstrates that 83.3% of people were sent to buy syringes from the pharmacy. On the other hand, only 16.6% of presenting patients did not need to buy syringes (Figure 3). In contrast, the second and third cycles showed a large percentage of people were supplied with equipment by the department for their presenting complaint. Cycles 2 and 3 demonstrated that 92.8% and 88.2% of patients did not have to buy syringes, which met our goal of provision of emergency care equipment (1 cc syringes) by 50% in two months’ time (Figures 4-5).

## Discussion

One of the most common causes of unilateral vision loss includes ocular trauma, which accounts for up to 8% of emergency department visits, of which 31% are cases of corneal foreign bodies [12]. The second most common cause of ocular trauma is corneal foreign bodies; the most common being corneal abrasions. Subconjunctival foreign bodies are a less common etiology of ocular trauma. Their extent of penetration is difficult to assess and may present as a foreign body granuloma [13]. A Swedish study revealed that corneal or conjunctival foreign bodies made up 40% of all eye injuries [14].

Sometimes, a foreign body may be lodged in the eye without the knowledge of the patient, and they may present with mild ocular symptoms. Usually, foreign bodies get lodged in the eye while working outdoors or doing a home improvement project. High-velocity injuries can occur in mechanics involved in hammering grinding and drilling. A study in Shenzhen, China, showed that males are more predisposed to ocular trauma due to their occupations [15]. Whenever a corneal foreign body is suspected, a history and ophthalmic examination are necessary. The mechanism of the injury must be investigated as well as the patient's occupation, the location of the event, and the usage of any protection at the time of injury must be inquired. Visual acuity and careful pupillary examination must be performed to rule out an intraocular foreign body as an abnormally shaped pupil or anisocoria may indicate otherwise [3].

It is recommended that foreign bodies should be removed from the eye as soon as possible, preferably in the first 24 hours, as a delay in achieving this could cause the foreign body to embed deeper into the cornea, making its removal harder [14]. Several methods of foreign body removal have been devised. Superficial foreign bodies can be washed with normal saline or can be removed with cotton-tipped applicators. A spud or 25-gauge syringe may be used to remove embedded substances. Patients should be referred to specialist care in case of complications like deeply embedded foreign bodies, corneal ulcerations, hyphema, hypopyon, or changes in visual acuity [12].

The use of disposable sterilized 1 cc syringes for the removal of foreign bodies can reduce the chances of infections involving the eye. It is also convenient if ophthalmic assessment rooms are stocked with such equipment, as patients do not need to purchase them. Furthermore, it may be quite troublesome for some patients, especially if they are alone, to buy instruments for their treatment, as foreign bodies can cause a lot of discomfort and irritation. Being pre-equipped can also avoid revisits to the pharmacy in case of contamination of a syringe. Our quality improvement project shows that the provision of syringes was improved to 92.8% and 88.2% in Cycles 2 and 3, from the previous statistics of 16.6%. This in turn improves patient satisfaction.

Superficial ulcers can heal by limbal stem cell migration, however, deep ulcers involve the stroma and hence need medical and surgical therapy for ulcers [16]. For minimal ocular surface defects, after removal of the foreign body, the patient is prescribed antibiotic eyedrops (for example, chloramphenicol) to be used for a few days to prevent infection. Alternatively, chloramphenicol ointment may be used. For patients with large abrasions, retained foreign bodies after irrigation, or contact lenses, levofloxacin eye drops may be prescribed [1]. For contact lens wearers, antibiotic drops sensitive to pseudomonas are used. Acetaminophen and ibuprofen may be used for pain relief. Topical steroids are usually avoided, as they delay wound healing. For patients who experience anterior chamber reaction, a short-acting topical cycloplegic agent can be used to reduce discomfort. Occasionally, a bandage contact lens may be used after the procedure to relieve pain and to reduce disruption associated with blinking. In order to improve healing and reduce scarring, anti-inflammatory therapy with amniotic membrane can be beneficial [3]. Preventative measures like eye covers during agricultural and mechanical activities must be adopted [11].

Metallic foreign bodies can lead to rust ring formation, which although rare can lead to reactive iritis. Foreign bodies that are superficial, peripheral, and have not penetrated the eye are usually associated with favorable outcomes [14]. Foreign body removal by unsterilized instruments, especially in the rural setups of developing countries can lead to ophthalmic infections like endophthalmitis [13].

The limitation, as in any low- or middle-income country (LMIC)-based tertiary care hospital is the approval of funding for maintaining the outcomes of the QIP over a sustained period of time. As this audit was conducted as a quality improvement project, if the changes are applied on a long-term basis, it can greatly improve patient satisfaction. This also includes improved feedback and a decrease in the number of complaints regarding patient care.

## Conclusions

Provision of emergency equipment is a basic right for anyone who presents with a complaint to the hospital emergency. We reached our goal of providing syringes for ocular foreign body removal for more than half of the patients who presented to us. This, in turn, increased patient satisfaction, as it improved the efficiency of emergency care and saved the patients' time and money.
